# An Adaptation and Validation Study of the Speech, Spatial, and Qualities of Hearing Scale (SSQ) in Italian Normal-Hearing Children

**DOI:** 10.3390/audiolres12030031

**Published:** 2022-05-29

**Authors:** Chiara Falzone, Letizia Guerzoni, Erica Pizzol, Enrico Fabrizi, Domenico Cuda

**Affiliations:** 1 Department of Otorhinolaryngology, “Guglielmo da Saliceto” Hospital, Via Cantone del Cristo 40, 29121 Piacenza, Italy; guerzoni1@libero.it (L.G.); e.pizzol@ausl.pc.it (E.P.); d.cuda@ausl.pc.it (D.C.); 2 Department of Economics and Social Sciences, Universita’ Cattolica del S. Cuore, Via Emilia Parmense 84, 29122 Piacenza, Italy; enrico.fabrizi@unicatt.it

**Keywords:** SSQ for children, SSQ for parents, subjective pediatric assessment, hearing loss, cochlear implant

## Abstract

This study aimed to translate and adapt the English version of the Speech, Spatial, and Qualities of Hearing Scale (SSQ) for children and for parents into the Italian language; validate SSQ for hearing children and their parents; and evaluate the discriminant validity of the instrument. A group of 102 normal-hearing Italian children, aged between 9 and 16 years, and their parents were included in this study. A group of 31 parents of normal-hearing Italian children aged between 6 and 8 years was also included. A group of 57 hearing-impaired Italian children aged between 9 and 16 years, and their parents were also included, as well as a group of 30 parents of hearing-impaired Italian children aged between 6 and 8 years. Cronbach’s alpha in the SSQ for parents was 0.92; it was 0.95 in the SSQ for children. Guttmann’s split-half coefficient in SSQ for children for both λ4 and λ6 was 0.98; in SSQ for parents in λ4 was 0.96 and λ6 was 0.95. These data provide evidence for the discriminant validity of the SSQ scale (*p*-value < 0.001). Italian SSQ scales for children and for parents are now available.

## 1. Introduction

The indications for a cochlear implant (CI) in children have increased [1,2,3,4] in recent years, and new objective and subjective assessment tools have been implemented [5,6].

Subjective inputs from patients and those who regularly interact with them are now included in the clinical protocols to determine the effectiveness and benefits derived from cochlear implants [7,8]. Furthermore, they are useful complements to assessment through objective testing.

Several assessment scales have been developed in the form of structured interviews with parents to provide professionals with information about the everyday listening situation of a child, especially for newborns, infants, and toddlers [9,10,11,12,13,14], while there are few assessment scales for school-aged children and adolescents [15,16,17,18].

Consequently, Galvin et al. [18] adapted the Speech, Spatial, and Qualities of Hearing Scale (SSQ), which was originally developed in adult clinical samples [19], for children, parents, and teachers. The original SSQ for adults was designed to evaluate the effects of a deficit in hearing in real-world listening environments. In particular, it is useful in obtaining information about speech perception in different conditions, spatial hearing, and other qualities of hearing [20].

SSQ is structured in three sections. Firstly, Section A (Speech) explores speech perception in different noise conditions, in groups, in reverberant environments, and in competing and dynamic speech listening settings. Secondly, Section B (Spatial hearing) evaluates the perception of direction, distance, and movement of sound sources. Finally, Section C (Other qualities of hearing) concerns recognition and segregation of sounds, ease of listening, identifiability of sounds, and naturalness/clarity.

Three versions were developed: one for children (33 items), one for parents (23 items), and one for teachers (21 items) [18,21,22].

The respondent is asked to use a 10-point visual analog scale (VAS), presented as a ruler, to rate their performance or experience in the listening scenario described.

Each scale score is computed as the mean of the scores associated with individual items. As a consequence, they range from 0 to 10.

Although pediatric SSQ has great utility potential, it has not been validated in the Italian language.

The aims of this study were to (1) translate into the Italian language and culturally adapt the English version of the Speech, Spatial, and Qualities of Hearing Scale (SSQ) for children and parents; (2) validate SSQ for hearing children and their parents; (3) evaluate the discriminant validity of the instrument.

## 2. Materials and Methods

### 2.1. Adaptation Procedure

Cross-cultural adaptation of the SSQ scales for both children and parents was performed using standard techniques [23]. The first step in validation involved a professional translator translating the questionnaire from English into Italian while ignoring the nature of SSQ for children and for parents. Then, this was back-translated from Italian to English by a mother-tongue bilingual speaker, who also had no previous knowledge of this tool. These versions of SSQ were submitted to three expert clinicians of the Department of Otorhinolaryngology, Head, and Neck Surgery, as well as 5 parents (3 males and 2 females) and 5 children (3 females and 2 males with a mean age of 11.2) in order to check their understanding of the questions. Lastly, some translation refinements were necessary so that the Italian version explores target behaviors similar to the original.

### 2.2. Study Design

This is a prospective, observational, nonrandomized study. All parents signed an informed consent form. The study was approved by the institutional local ethics committee (Prot. No. 177/2019/OSS/AUSLPC).

### 2.3. Participants

#### 2.3.1. Population with Normal Hearing

A group of 102 normal-hearing Italian children (77 females, 55 males) aged between 9 and 16 years (mean 10.6) (Table 1) and their parents were included in this study. 

An additional group of 31 parents of normal-hearing Italian children (9 females, 22 males) aged between 6 and 8 years (mean 7.5) (Table 1) was also included.

The normal-hearing children were recruited from local primary, middle, and high schools in both rural and urban areas. 

The pediatrician of recruited children was asked to fill in an ad hoc questionnaire about any hearing disorder of the child (sensorineural or conductive hearing loss), as well as any audiological risk factors for hearing impairment according to the Joint Committee on Infant Hearing [24]. If a report was abnormal, the child was excluded from the study. Therefore, four children were excluded; three had educational disorders, and one had CMV infection at birth. Furthermore, exclusion criteria also included neurodegenerative disorders, educational disorders, and non-native speaking parents.

#### 2.3.2. Hearing-Impaired Population

A group of 54 hearing-impaired Italian children (30 females, 24 males) aged 9–16 years (mean 12) (Table 1) and their parents were included in this study.

An additional group of 23 parents of hearing-impaired Italian children (13 females, 10 males) aged 6–8 years (mean 6.8) (Table 1) was also included.

Children were recruited from the audiological service of our department.

Inclusion criteria were composed of the presence of sensorineural hearing loss (SNHL) with a pure tone average at 500–4000 Hz greater than 35 dB in the best year. The children used different types of hearing devices: 19 used hearing aids (HA) bilaterally, 16 wore a unilateral cochlear implant (CI), 20 had bilateral CI, and 22 used bimodal stimulation (CI in one ear and HA in the unimplanted ear). 

### 2.4. Questionnaire Administration

#### 2.4.1. SSQ for Parents

The questionnaire was given to the parents by an experienced speech therapist. Considering that some listening scenarios could be difficult to evaluate, an observation period of one week for each section was required, as suggested by Galvin and Noble [18]. Therefore, we provided parents with a list of the listening scenarios to help them in the observation of their child.

The SSQ was finally administered to parents by an experienced speech therapist through a telephone interview. 

#### 2.4.2. SSQ for Children

The questionnaire was administered by an experienced speech therapist in a face-to-face interview in the school of the child.

The Italian versions of the SSQ for parents and for children are shown in Supplementary Materials File S1.

### 2.5. Statistical Analysis

As expected, there were some unanswered items, and the mean age of respondents whose questionnaire had at least one missing item was compared with those without any. In addition, the *t*-test and Wilcoxon test were used.

Cronbach’s alpha was used to assess the internal consistency of the scale. Guttman’s reliability indexes (λ4 and λ6) were also considered [25]. Furthermore, the item-level statistics were also computed—namely, means, standard deviation, and item-to-rest correlations.

The discriminant validity of the scale was assessed by comparing the scores of normal-hearing children with those of hearing-impaired children. Therefore, a Wilcoxon signed-rank test for independent samples was computed for this purpose. In principle, a two-sample *t*-test could be used, but normality tests (Shapiro–Wilks) led to the null hypothesis being rejected in all cases [26]. Effect sizes computed were also considered. Both the whole scales and each of the subscales were analyzed. The correlation between the age of the child and score on the SSQ scale was assessed using a Spearman correlation because of non-normality. The correlations (Pearson’s) between the children’s scores and their parents’ scores for the scores associated with the three-section subscales were computed.

The R software was used for all statistical computations [27]. Specifically, internal consistency and scale reliability statistics were obtained using functions in the psych package [28].

## 3. Results

Some missing data were found; they ranged from 1% to 10% (mean 3%) in SSQ for parents. A high percentage of missing data was found in items 5 and 6 of Section B.

The range of missing data in SSQ for children was 1–10% (mean 2.3%). The largest volume of missing data was found in item 13 of Section B. The detailed distribution is shown in Supplementary Materials File S2.

### 3.1. Internal Consistency

Cronbach’s alpha for the SSQ score for children was 0.96, and Cronbach’s alpha for the SSQ score for parents was 0.93 [29].

### 3.2. Scale Reliability

Guttmann’s split-half coefficient for both λ4 and λ6 in SSQ for children was 0.98.

Guttmann’s split-half coefficient in SSQ for parents was 0.96 for λ4 and 0.95 for λ6.

### 3.3. Item Reliability

Analysis shows that there were no single problematic items since they all showed positive correlations with the overall score, even though some items performed much better than others. Items with lower correlation were the following: in SSQ, for parents item 7 in Section C; in SSQ for children item 2 in Section A, item 13 in Section B, and items 3, 4, 6, 9 in Section C Correlations ranged from 0.3 to 0.8, with few exceptions. All correlations were significant (*p*-values < 0.001) and are shown in Supplementary Materials File S3.

### 3.4. Discriminant Validity

Discriminant validity was assessed by comparing the SSQ scores obtained for the sample of normally hearing children to those computed on the sample of hearing-impaired children. The questionnaires filled in by the children and their parents were compared separately.

The results of the Wilcoxon tests are reported in Table 2 and Table 3 They show that the null hypotheses of equal distributions in the two populations have to be rejected (*p*-values < 0.001).

Although normality tests (Shapiro–Wilks) led to the rejection of the null hypothesis in all cases [26], a two-sample *t*-test comparing the means could also be used, because of the relatively large sample size. These results are not presented here, but they are in line with those shown.

The effect sizes were computed according to the qualitative scale suggested by Tomczak and Tomczak [30].

**Figure 1:**
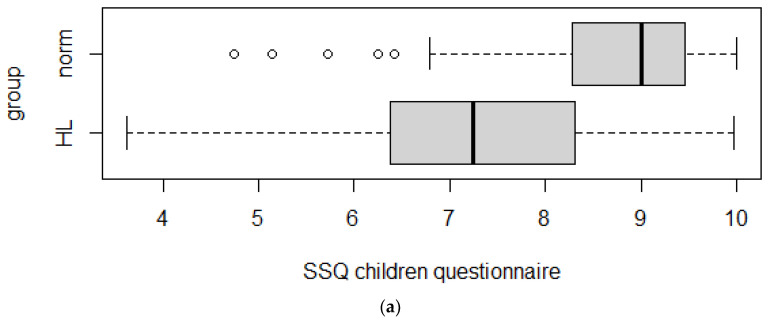

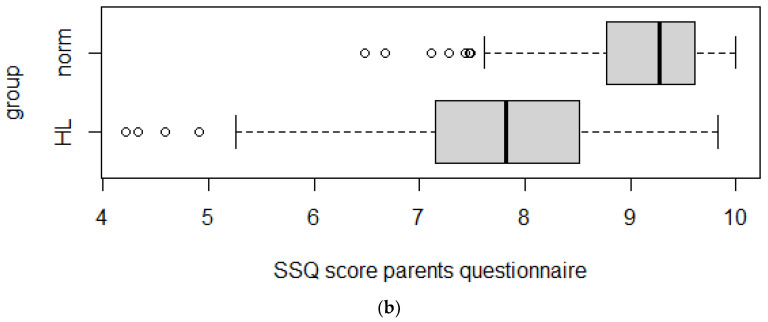
Boxplots of SSQ for children and for parents total scores in normal-hearing and hearing-impaired children. (**a**): SSQ children questionnaire; (**b**): SSQ score parents questionnaire.

All of the effects found were large. The analysis was complemented by adding the plots in Figure 1.

Supplementary Materials File S4 shows the discriminant validity of the SSQ score for the different subscales. Discriminant validity was also confirmed at the level of subscales.

### 3.5. Analysis of the Impact of Age of Children on Answers

No significant was found for the questionnaire filled in by parents. In fact, the correlation was very close to 0 (0.002), and the *p*-value was 0.96.

**Figure 2:**
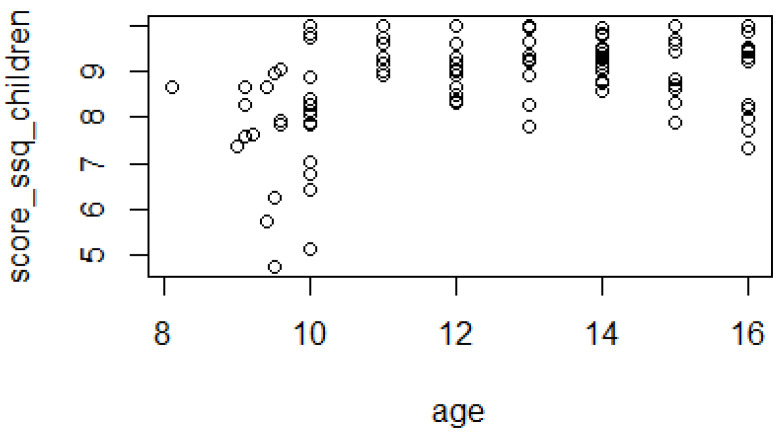
Total SSQ for children’s score as a function of age.

On the other hand, the correlation was 0.374 for questionnaires filled in by children and different from 0, which was statistically significant (*p*-value 0.003). The relationship was driven by the higher frequency of lower score values for young children aged 10 or below (Figure 2).

On the contrary, the correlation in the subsample of children aged 11 or older (74 cases out of 102) was negative (−0.19) and not statistically significant (*p*-value 0.11).

As a further analysis, the sample of children completing the questionnaire and completing an ANOVA type of analysis was divided.

**Figure 3:**
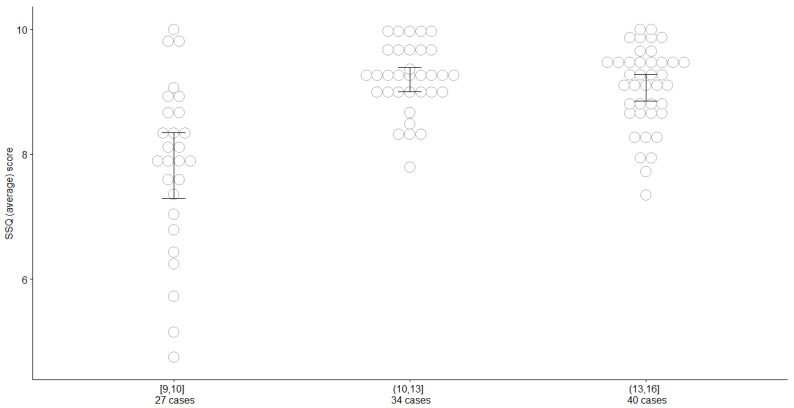
Individual scores are displayed in grey (ties are shown side by side). Black bars represent 95% confidence intervals. The one-way ANOVA has an associated *p*-value. It can be seen that the first group (younger pupils) is different from the other two, which are not different on average.

Specifically, the age was first divided into three groups—namely [9,10,11,13,14,16]. The hypothesis of normality was not rejected for the SSQ in each of the three groups, so means could be compared using a one-way ANOVA test. The results are summarized in Figure 3.

### 3.6. Correlations between the Children’s Scores and Their Parents’ Scores

All correlations were quite low. Nevertheless, correlations were statistically significant.

- Section A: 0.28 (*p*-value 0.03);
- Section B: 0.38 (*p*-value < 0.001);
- Section C: 0.22 (*p*-value 0.03).

The moderating effect of age was considered through a linear model specified by regressing the score obtained from questionnaires filled in by parents on the one obtained from the questionnaire filled in by children. In all cases, age has a moderating and statistically significant role. The relationship between parents and children scores remained statistically significant. Data are shown in Supplementary Materials File S5.

## 4. Discussion

The main aim of this study was to adapt and validate a version of the SSQ scales for parents and for children in the Italian language. The procedure involved a back translation requiring various steps and refinements so that the Italian version would explore similar target behaviors as the original.

As already noted in the literature, some of the data for some of the items in the preliminary analysis of data were found to be missing [31]. In SSQ for parents, higher percentages of missing data were found in Section B: items 5, 6 (“*You are talking with your child. There is a continuous background noise, such as a fan or running water. Can your child follow what you say?”; ” Your child is in a group of about five people, sitting round a table. It is a noisy room, such as a busy restaurant or large family gathering at home. Your child cannot see everyone else in the group. Can your child follow the conversation?*”). This could be explained by the fact that, despite the given observation period, there are situations, especially with spatial hearing, that are difficult for parents to observe and consequently evaluate [32].

In SSQ for children, a higher percentage of missing data was found in Section B: item 13 (“Do the things you can hear seem to be inside your head rather than outside in the world? For example, if you can see a dog barking across the street, does it sound to you like the dog is across the street or does it seem to be inside your head?”). Since the questionnaire was administered in a face-to-face interview, we observed that this was the most difficult item for children to understand, despite the explanation given.

The final version demonstrated good general internal consistency, good scale reliability, and item reliability (*p*-values < 0.001). Some items were much better performing than others. The item with the lowest correlation in SSQ for parents is item 7 in Section C (“*Does your child have to put in a lot of effort to hear what is being said in conversation with others?*”). The authors believe that it could be difficult for a parent to quantify the listening effort made by a child.

Different items have lower correlations in SSQ for children.

Concerning item 2 in Section A (*“You are talking with your child in a quiet, carpeted lounge-room. Can your child follow what you’re saying?”*), the reference to a “carpeted lounge-room” was already reported to be a fairly uncommon listening situation, and the low correlation could be a problem of item equivalence [19,33,34].

Item 13 in Section B (“Do the things you can hear seem to be inside your head rather than outside in the world? For example, if you can see a dog barking across the street, does it sound to you like the dog is across the street, or does it seem to be inside your head?”) has a lower correlation and, as mentioned above, a higher missing data percentage. This may depend on the complexity of the item and its construct being different from the others in the same section.

The lower correlation in the case of items 3, 4, 6, and 9 in Section C (“Do you know which person in your family is talking just by the sound of their voice, even if you cannot see them?”; “You can hear a song you know being played. Is it easy for you to tell what song it is just by listening?”; “Can you tell how someone feels (happy, angry, sad) just by listening to their voice?”; “Do you have to try hard to understand what other people are saying?”) could be explained by the fact that they refer to easier listening situations for normal-hearing children than the others in the same section.

Furthermore, in order to assess the degree to which the SSQ questionnaire measures the construct it is supposed to measure, the SSQ score of normal-hearing children was compared with the SSQ score of hearing-impaired children.

The data obtained provided positive evidence of the discriminant validity of the SSQ scale. Discriminant validity was also confirmed at the level of subscales.

This means that the proposed SSQ adaptation has satisfactory psychometric characteristics because it enjoys both reliability and the ability to discriminate between populations that are known in advance to be different in terms of the construct being measured.

Finally, the impact of the age of the children on the SSQ score was analyzed. No significant correlation with the questionnaire filled in by parents was found.

Conversely, questionnaires filled in by children showed an age effect. In particular, children younger than 10 years scored lower than the older ones. These results raise the problem of the minimum age of administration.

Galvin et al. did not administer the scale to any children with hearing loss younger than 11 years and feel that the reliability of the self-ratings may be reduced for younger children [18]. This topic is controversial since there are a number of factors to consider: the respondent, the number and type of scenarios in which performance is being measured, and the complexity of the response format. In fact, other studies of assessment procedures for pediatric hearing aid fitting outcomes have indicated that children between 8 and 10 years of age can provide useful information [15]. For example, the recommended minimum age of administration for the children’s home inventory for listening difficulties is 7–8 years [16].

In clinical practice, the authors have found that children aged 9 years and above have good reading skills and enough cognitive maturity to provide useful information about their hearing abilities in everyday life [35]. As a result, children 9–16 years of age were included in the SSQ for children administered in the study presented in this paper. Regarding the SSQ for parents, in line with Galvin et al. [18], it was decided to include parents of children between 6 and 16 years of age [36]. Consequently, SSQ for children between 9 and 16 years of age and SSQ for parents of children aged between 6 and 16 years were validated.

In light of these considerations and given the results, it is valid to administer the questionnaire in the age group selected, paying particular attention to children under 10 years of age.

The pediatric scales for children, as well as parents and teachers, were adapted from Galvin et al. (2013). As far as the authors know, this Italian validation of the SSQ for parents is the first to be published in the literature. Moreover, SSQ for children and adolescents has also only recently been translated and validated in the Dutch language [31].

The parental versions of SSQ are used in various studies investigating auditory benefits in cochlear implanted children [37,38,39,40,41]. There are fewer studies in which SSQ for children is used, and they relate to children with unilateral hearing loss [35,36].

The significant differences that were found between hearing=impaired and normal-hearing children suggest that these scales could be useful in the assessment of hearing abilities in deaf children.

SSQ scales could be included in the follow-up evaluation tools and be used as a potential screening instrument, although further research should be carried out for this purpose.

Furthermore, the results demonstrate that parents and children themselves are valid and important sources of information and that using questionnaires is a pragmatic solution.

A reliable Italian adaptation of SSQ scales for children and for parents is now available. Further research needs to be carried out to validate the clinical use of these instruments.

## Figures and Tables

**Table 1:** Normal hearing and hearing impaired children per age.

Age of Children (Years)	Normal-Hearing Children (No.)	Hearing-Impaired Children (No.)
6	7	12
7	12	4
8	12	7
9	14	3
10	14	9
11	11	11
12	15	8
13	9	6
14	17	10
15	11	3
16	11	4

**Table 2:** SSQ for parents and children in the normal-hearing and hearing-impaired children samples.

SSQ	Normal Hearing (Median)	Hearing Impaired (Median)	*p*-Value	Effect Size	Effect Size Evaluation
For parents	9.28	7.80	<0.001	0.64	Large
For children	9.00	7.09	<0.001	0.55	Large

**Table 3:** SSQ subscales for parents and children in the normal-hearing and hearing-impaired children samples.

Scale	Normal Hearing (Median)	Hearing Impaired (Median)	*p*-Value	Effect Size	Effect Size Evaluation
Section A (parents)	9.22	7.78	<0.001	0.59	Large
Section A (children)	9.00	7.30	<0.001	0.53	Large
Section B (parents)	9.17	7.33	<0.001	0.58	Large
Section B (children)	8.85	6.89	<0.001	0.47	Moderate
Section C (parents)	9.38	8.12	<0.001	0.48	Moderate
Section C (children)	9.40	7.94	<0.001	0.50	Large

## Data Availability

Not applicable.

## Supplementary Materials

The following supporting information can be downloaded at: https://www.mdpi.com/article/10.3390/audiolres12030031/s1.
