# Supplementary material for: An Adaptation and Validation Study of the Speech, Spatial, and Qualities of Hearing Scale (SSQ) in Italian Normal-Hearing Children
Source: Audiol Res. 2022 May 29;12(3):297–306. doi: 10.3390/audiolres12030031 (PMC9220328; doi:10.3390/audiolres12030031)
Supplement: Supplementary file 1 [file audiolres-12-00031-s001.zip › audiolres-1642444-supplementary/supplementary materials file S1.pdf]

Questionario di Percezione del Parlato, Spaziale e Qualità di Ascolto (SSQ)

per genitori di bambini con Dispositivi Acustici

(Basato sul questionario SSQ per adulti sviluppato da William Noble & Stuart Gatehouse;

modificato da Karyn Galvin)

Sezione A: Percezione del parlato

1. Lei sta parlando con il suo bambino e c'è la TV nella stessa stanza. Senza abbassare la TV, il bambino riesce a seguire quello che sta dicendo?
2. Lei sta parlando con il suo bambino in un salottino silenzioso. Il bambino riesce a seguire quello che sta dicendo?
3. Il bambino è in un gruppo di cinque persone, sedute attorno ad un tavolo. È un luogo tranquillo, può vedere ogni persona del gruppo. Riesce a seguire la conversazione?
4. Il bambino è in un gruppo di cinque persone, sedute attorno ad un tavolo. È una stanza rumorosa, come un ristorante affollato o un grande ritrovo di famiglia. Il bambino può vedere ogni persona del gruppo. Riesce a seguire la conversazione?
5. Lei sta parlando con il suo bambino. C'è un continuo rumore di sottofondo, come un ventilatore o l'acqua che scorre. Il bambino riesce a seguire quello che sta dicendo?
6. Il bambino è in gruppo di cinque persone, sedute attorno ad un tavolo. È una stanza rumorosa, come un ristorante affollato o un grande ritrovo di famiglia a casa, non può vedere le persone del gruppo. Il bambino riesce a seguire la conversazione?
7. Lei stai parlando con il suo bambino in un posto dove ci sono molti echi, come in una sala di assemblea scolastica o all'interno di una piscina. Il bambino può seguire quello che sta dicendo?
8. Lei sta parlando con il suo bambino in una stanza in cui ci sono molte altre persone che parlano. Il bambino riesce a seguire quello che sta dicendo?
9. Suo figlio riesce ad avere facilmente una conversazione al telefono con una persona familiare?

Sezione B: Percezione spaziale

1. Il suo bambino è all'aperto in un luogo non familiare. È possibile udire un forte rumore costante, ad esempio da un tosaerba, un aereo o uno strumento elettrico. La fonte del suono non può essere vista. Il bambino è in grado di capire immediatamente da dove proviene il suono?

2. Il suo bambino è seduto attorno ad un tavolo con diverse persone. Non può vedere nessuno. Il bambino è in grado di dire dove si trova una persona non appena essa inizia a parlare?
3. Il suo bambino è seduto tra te e un'altra persona. Uno di voi inizia a parlare. Il bambino è in grado di capire immediatamente se a parlare è la persona alla sua sinistra o alla sua destra senza dover guardare?
4. Lei e il suo bambino siete a casa in stanze diverse. C'è quiete. Se suo figlio sente chiamare il suo nome, saprà in quale parte della casa lei si trova?
5. Il suo bambino è all'aperto Un cane abbaia. Il bambino riesce a dire immediatamente dove si trova, senza dover guardare?
6. Il suo bambino si trova sul marciapiede di una strada trafficata. Il bambino riesce a dire immediatamente da quale direzione arriva un autobus o un camion prima di vederlo?

Sezione C: Qualità della percezione

1. Pensi a quando ci sono due rumori dentro o intorno alla casa contemporaneamente, per esempio, acqua corrente dentro la vasca da bagno e una radio che suona, o un camion che passa e il suono di bussare alla porta. Il bambino è in grado di identificarli come due suoni separati?
2. Lei è in una stanza con il bambino e c'è della musica. Il bambino sarà in grado di identificare la voce se lei inizia a parlare? Notare che il tuo bambino non deve capire quello che lei sta dicendo.
3. Il suo bambino riesce a riconoscere membri della famiglia o altre persone a lui familiari dal solo suono della voce, senza vederli?
4. Il suo bambino riesce a distinguere differenti brani musicali, familiari? Notare che produrre parole o movimenti relativi ad una canzone può indicarne il riconoscimento.
5. Il suo bambino riesce a dire la differenza tra suoni che sono piuttosto simili, per esempio, una macchina rispetto ad un bus, o acqua che bolle in una pentola rispetto a del cibo che cuoce in padella?
6. Il suo bambino riesce a giudicare facilmente l'umore delle persone dal suono della voce?
7. Suo figlio deve impegnarsi molto per sentire quello che viene detto in una conversazione con altre persone?
8. Il suo bambino riesce facilmente ad ignorare alcuni suoni quando sta cercando di ascoltare qualcosa?

Questionario di Percezione del Parlato, Spaziale e Qualità di Ascolto (SSQ)

per Bambini con Dispositivi Acustici

(Basato sul questionario SSQ per adulti sviluppato da William Noble & Stuart Gatehouse;

modificato da Karyn Galvin)

Sezione A: Percezione del parlato

1. Stai parlando con tua mamma o con tuo papa e nella stessa stanza c'è la TV. Senza abbassare il volume della TV, riesci a capire cosa stanno dicendo?
2. Stai parlando con una persona in un salottino silenzioso, riesci a capire cosa sta dicendo l'altra persona?
3. Sei in un gruppo di cinque persone, sedute attorno ad un tavolo. È una situazione di quiete. Tu puoi vedere tutte le persone del gruppo, riesci a capire di cosa si sta parlando nel gruppo?
4. Sei in un gruppo di cinque persone, sedute attorno ad un tavolo. È una stanza rumorosa, dove ci sono studenti che si muovono e parlano. Puoi vedere tutti nel gruppo attorno al tavolo, riesci a capire di cosa si sta parlando?
5. Stai parlando con una persona, c'è rumore di sottofondo, come un rubinetto in funzione o un ventilatore. Riesci a capire quello che la persona ti sta dicendo?
6. Sei in un gruppo di cinque persone, sedute attorno ad un tavolo. È una stanza rumorosa, dove ci sono studenti che si muovono e parlano. Non puoi vedere tutti quelli del gruppo attorno al tavolo, riesci a capire quello di cui sta parlando il gruppo?
7. Stai parlando con qualcuno in un luogo dove ci sono molti echi, come un'aula di assemblea scolastica, riesci capire quello che stanno dicendo le persone?
8. Stai parlando con una persona in una stanza in cui ci sono altre persone che parlano, riesci a capire quello che stanno dicendo le altre persone?
9. Stai parlando con un gruppo di amici e tutti stanno parlando a turno. Riesci a capire ogni volta che una persona comincia a parlare (senza perdere il discorso)?
10. È facile per te parlare al telefono con un amico o tua mamma o tuo papà?

Sezione B: Percezione spaziale

1. Sei fuori in un posto dove non sei mai stato prima. C'è un forte rumore proveniente da un tosaerba o un aeroplano che non puoi vedere, riesci a capire subito da dove proviene il suono?
2. Sei in gruppo di cinque persone, sedute attorno ad un tavolo. Non puoi vedere tutti quelli del gruppo, riesci a capire dove si trova qualcuno appena inizia a parlare?
3. Sei seduto tra due amici, uno di questi inizia a parlare. Senza guardare riesci subito ad individuare se è l'amico di destra o sinistra che parla?

4. Sei a casa e c’è quiete. Tua mamma o tuo papà ti chiamano da un’altra stanza. Riesci a capire dove sono?

5. Sei all’aperto, un cane che abbaia forte. Senza cercarlo con lo sguardo, riesci a dire velocemente dove si trova?
6. Sei sul marciapiede di una strada trafficata. Riesci a sentire un autobus o un camion e a capire subito da dove proviene prima di vederlo?
7. Stai sentendo un autobus o un camion. Riesci a capire se si sta avvicinando a te o si sta allontanando, solo dal suono (senza guardare)?
8. Stai sentendo voci e passi. Riesci a capire se la persona viene verso di te o si allontanando, solo dal suono (senza guardare)?
9. Stai sentendo un autobus o un camion. Riesci a capire in quale direzione si sta muovendo (ad esempio, da sinistra a destra o da destra a sinistra) solo dal suono, senza guardare?
10. Stai sentendo voci o passi. Riesci a capire in quale direzione si sta muovendo la persona (ad esempio, da sinistra a destra o da destra a sinistra) solo dal suono, senza guardare?
11. Stai sentendo un autobus o un camion. Riesci a capire quanto è lontano dal suono?
12. Stai sentendo voci o passi. Riesci a capire quanto è lontana la persona dal suono?
13. Le cose che riesci a sentire sembrano essere nella tua testa piuttosto che fuori nel mondo? Ad esempio, se riesci a vedere un cane che abbaia dall'altra parte della strada, ti sembra che il cane sia dall'altra parte della strada o sembra che si trovi nella tua testa?

Sezione C: Qualità della percezione

1. Sei in una stanza in cui c’è musica. Qualcuno comincia a parlare, riesci a capire se qualcuno ha iniziato a parlare (anche se non sai quello che sta dicendo)?
2. Pensa a quando senti due rumori contemporaneamente, ad esempio, l'acqua che scorre nella vasca da bagno e una radio, o un camion che passa e il suono di bussare alla porta. Li senti come due suoni separati?
3. Riesci a riconoscere solo dalla voce chi sta parlando della tua famiglia?
4. Quando ascolti una canzone che conosci, riesci a riconoscerla?
5. Riesci a riconoscere la differenza tra rumori simili tra loro come per esempio, una macchina rispetto ad un autobus, o rubinetto che scorre e un ventilatore?
6. Riesci a capire come si sente qualcuno (felice, arrabbiato, triste) solo ascoltando la sua voce?
7. Fai molta fatica quando ascolti qualcuno o qualcosa?
8. Quando stai viaggiando sul sedile anteriore della macchina riesci a capire facilmente quello che l’autista ti sta dicendo?
9. Fai fatica a capire quello che dicono le altre persone?
10. Per te è facile escludere altri suoni quando provi ad ascoltare qualcuno?
