# Supplementary material for: An Adaptation and Validation Study of the Speech, Spatial, and Qualities of Hearing Scale (SSQ) in Italian Normal-Hearing Children
Source: Audiol Res. 2022 May 29;12(3):297–306. doi: 10.3390/audiolres12030031 (PMC9220328; doi:10.3390/audiolres12030031)
Supplement: Supplementary file 1 [file audiolres-12-00031-s001.zip › audiolres-1642444-supplementary/supplementary materials file S2.pdf]

Please find below a detailed analysis of the number of missing responses for each of the item.

Section A (parent)

Item	1	2	3	4	5	6	7	8	9
Fully compiled	131	133	133	131	131	127	132	131	133
Missing	2	0	0	2	2	6	1	2	0

Section B (parent)

Item	1	2	3	4	5	6
Fully compiled	126	127	132	131	124	121
Missing	7	6	1	2	9	12

Section C (parent)

Item	1	2	3	4	5	6	7	8
Fully compiled	126	130	133	132	125	133	127	128
Missing	7	3		1	8		6	5

Section A (child)

Item	1	2	3	4	5	6	7	8	9	10
Fully compiled	101	99	100	98	100	98	101	100	100	100
Missing	1	3	2	4	2	4	1	2	2	2

Section B (child)

Item	1	2	3	4	5	6	7	8	9	10	11	12	13
Fully compiled	100	99	101	101	99	98	101	100	98	100	98	100	93

Missing	2	3	1	1	3	4	1	2	4	2	4	2	9

Section C (child)

Item	1	2	3	4	5	6	7	8	9	10
Fully compiled	101	99	100	98	100	98	101	100	100	100
Missing	1	3	2	4	2	4	1	2	2	2
