# Supplementary material for: An Adaptation and Validation Study of the Speech, Spatial, and Qualities of Hearing Scale (SSQ) in Italian Normal-Hearing Children
Source: Audiol Res. 2022 May 29;12(3):297–306. doi: 10.3390/audiolres12030031 (PMC9220328; doi:10.3390/audiolres12030031)
Supplement: Supplementary file 1 [file audiolres-12-00031-s001.zip › audiolres-1642444-supplementary/supplementary materials file S3.pdf]

SSQ for parents. Item level statistics based on the 102 questionnaires without missing items

Section	item	mean	sd	item-rest correlation
A	1	9.0	1.33	0.66
A	2	9.7	0.72	0.49
A	3	9.5	0.77	0.60
A	4	8.7	1.19	0.68
A	5	9.2	0.98	0.67
A	6	8.3	1.50	0.65
A	7	8.6	1.40	0.70
A	8	8.7	1.31	0.62
A	9	9.6	0.89	0.59
B	1	8.8	1.42	0.59
B	2	8.8	1.32	0.45
B	3	9.6	0.82	0.56
B	4	9.2	1.00	0.59
B	5	8.9	1.18	0.64
B	6	8.8	1.37	0.58
C	1	9.5	0.76	0.66
C	2	9.4	0.98	0.66
C	3	9.7	0.60	0.62
C	4	9.5	0.82	0.71
C	5	9.1	1.16	0.54
C	6	9.2	0.95	0.44
C	7	8.9	1.82	0.38
C	8	8.5	1.53	0.62

SSQ for children. Item level statistics based on the 89 questionnaires without missing items

Section	item	mean	sd	item-rest correlation
A	1	8.9	1.51	0.61
A	2	9.6	0.98	0.38
A	3	9.1	1.21	0.57
A	4	8.0	1.96	0.86
A	5	9.3	1.03	0.61
A	6	7.8	2.09	0.80
A	7	8.5	1.55	0.65
A	8	8.1	1.78	0.66
A	9	9.4	1.11	0.49
A	10	9.6	0.78	0.40
B	1	8.3	1.94	0.77
B	2	8.6	1.64	0.75
B	3	9.2	1.37	0.73

B	4	9.0	1.28	0.63
B	5	8.5	1.48	0.83
B	6	8.6	1.42	0.70
B	7	8.8	1.42	0.70
B	8	8.7	1.44	0.77
B	9	8.4	1.56	0.68
B	10	8.3	1.59	0.74
B	11	8.6	1.27	0.63
B	12	8.5	1.35	0.60
B	13	9.0	1.50	0.29
C	1	9.0	1.47	0.73
C	2	9.0	1.65	0.76
C	3	9.7	0.59	0.19
C	4	9.7	0.62	0.27
C	5	9.0	1.41	0.69
C	6	9.2	0.91	0.37
C	7	9.4	0.83	0.40
C	8	9.3	0.88	0.40
C	9	9.5	0.65	0.37
C	10	8.8	1.63	0.64
