# Supplementary material for: An Adaptation and Validation Study of the Speech, Spatial, and Qualities of Hearing Scale (SSQ) in Italian Normal-Hearing Children
Source: Audiol Res. 2022 May 29;12(3):297–306. doi: 10.3390/audiolres12030031 (PMC9220328; doi:10.3390/audiolres12030031)
Supplement: Supplementary file 1 [file audiolres-12-00031-s001.zip › audiolres-1642444-supplementary/supplementary materials file S5.pdf]

```
SECTION A
Coefficients:
              Estimate Std. Error t value Pr(>|t|)    
(Intercept)    7.65096    0.66842  11.446  < 2e-16 ***
score_gi       0.28867    0.07668   3.765 0.000282 ***
age           -0.08802    0.03645  -2.415 0.017559 *  
---
Signif. codes:  0 ‘***’ 0.001 ‘**’ 0.01 ‘*’ 0.05 ‘.’ 0.1 ‘ ’ 1
```

```
SECTION B
Coefficients:
              Estimate Std. Error t value Pr(>|t|)    
(Intercept)    7.73559    0.57986  13.340  < 2e-16 ***
score_gii      0.32661    0.06411   5.095 1.67e-06 ***
age           -0.11215    0.03366  -3.332  0.00121 ** 
---
Signif. codes:  0 ‘***’ 0.001 ‘**’ 0.01 ‘*’ 0.05 ‘.’ 0.1 ‘ ’ 1
```

```
SECTION C
Coefficients:
              Estimate Std. Error t value Pr(>|t|)    
(Intercept)    7.92576    0.70947  11.171  < 2e-16 ***
score_giii     0.27544    0.08420   3.271  0.00147 ** 
datiopSSQ$age -0.09379    0.03188  -2.942  0.00405 ** 
---
Signif. codes:  0 ‘***’ 0.001 ‘**’ 0.01 ‘*’ 0.05 ‘.’ 0.1 ‘ ’ 1
```
