# Supplementary figures and images for: An Adaptation and Validation Study of the Speech, Spatial, and Qualities of Hearing Scale (SSQ) in Italian Normal-Hearing Children
Source: Audiol Res. 2022 May 29;12(3):297–306. doi: 10.3390/audiolres12030031 (PMC9220328; doi:10.3390/audiolres12030031)

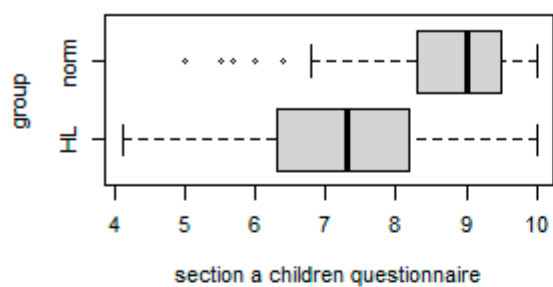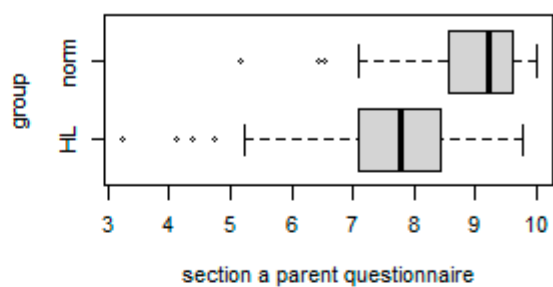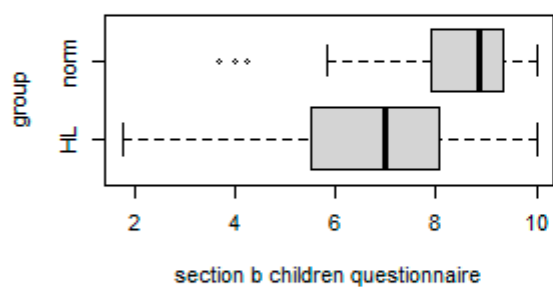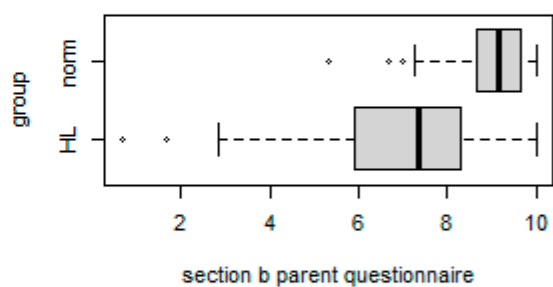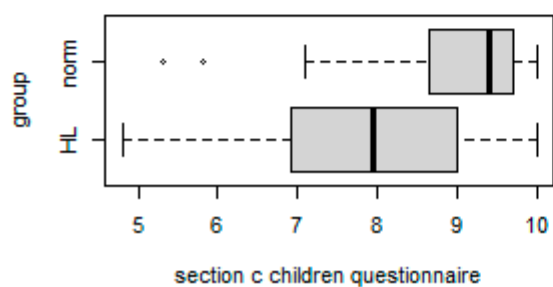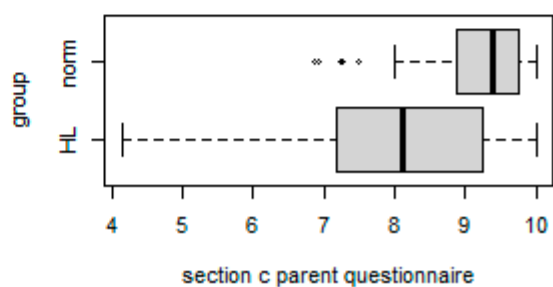

Supplement: Supplementary file 1 [file audiolres-12-00031-s001.zip › audiolres-1642444-supplementary/supplementary materials file S4.pdf]
